# Clients of sex workers in Switzerland: it makes sense to counsel and propose rapid test for HIV on the street, a preliminary report

**DOI:** 10.1186/1471-2334-10-74

**Published:** 2010-03-19

**Authors:** Esther-Amélie Diserens, Patrick Bodenmann, Chantal N'Garambe, Anne Ansermet-Pagot, Marco Vannotti, Eric Masserey, Matthias Cavassini

**Affiliations:** 1 Department of Ambulatory Care and Community Medicine, University of Lausanne, Lausanne, Switzerland; 2 Association Fleur de Pavé, Lausanne, Switzerland; 3 Department of Public Health, Lausanne, Vaud, Switzerland; 4 Service of Infectious Diseases, Centre Hospitalier Universitaire Vaudois, University of Lausanne, Lausanne, Switzerland

## Abstract

**Background:**

Clients of street sex workers may be at higher risk for HIV infection than the general population. Furthermore, there is a lack of knowledge regarding HIV testing of clients of sex workers in developed countries.

**Method:**

This pilot study assessed the feasibility and acceptance of rapid HIV testing by the clients of street-based sex workers in Lausanne, Switzerland. For 5 evenings, clients in cars were stopped by trained field staff for face-to-face interviews focusing on sex-related HIV risk behaviors and HIV testing history. The clients were then offered a free anonymous rapid HIV test in a bus parked nearby. Rapid HIV testing and counselling were performed by experienced nurse practitioners. Clients with reactive tests were offered confirmatory testing, medical evaluation, and care in our HIV clinic.

**Result:**

We intercepted 144 men, 112 (77.8%) agreed to be interviewed. Among them, 50 (46.6%) had never been tested for HIV. A total of 31 (27.7%) rapid HIV tests were performed, 16 (51.6%) in clients who had not previously been tested. None were reactive. Initially, 19 (16.9%) additional clients agreed to HIV testing but later declined due to the 40-minute queue for testing.

**Conclusion:**

This pilot study showed that rapid HIV testing in the red light district of Lausanne was feasible, and that the clients of sex workers accepted testing at an unexpectedly high rate. This setting seems particularly appropriate for targeted HIV screening, since more than 40% of the clients had not previously been tested for HIV even though they engaged in sex-related HIV risk behaviour.

## Background

Since 1999, a yearly 3- to 5-evening preventive action called "Don Juan" has taken place on the streets of the red light districts in major Swiss cities. Financed by l'Aide Suisse contre le SIDA (Swiss help foundation against AIDS), this event aims to increase AIDS and STD (Sexually Transmitted Disease) awareness among the clients of sex workers.

The prevalence of HIV in sex workers in Switzerland is unknown; however, the prevalence is presumed to be higher than in the general population due to multiple risk factors. First, sex workers are a very heterogeneous group that includes forced migrants, injecting drug users, transsexuals, and individuals from other vulnerable and at-risk populations [1]. Furthermore, the sex workers often have unstable living and working situations. As a consequence, their access to healthcare and disease prevention information is generally not optimal compared to the general population [2-4].

The prevalence of HIV in the clients of sex workers in Switzerland is also unknown. This lack of knowledge stems from the difficulty of contacting clients in western countries; in addition, there is little epidemiological information about men who pay for sex [4,5]. However, previous studies in Kenya, Benin, China, Peru and India have reported a high prevalence of HIV infection, STDs, and risk behaviours among clients of sex workers; thus, such clients could serve as a bridge population for HIV/STD transmission [6-12].

The 2007 Don Juan local report found that about 40% of clients had never taken an HIV test [13], either because they feared a positive result or because they didn't consider themselves to be at risk [14]. However, sex workers report that clients frequently requested unprotected sex (mainly oral intercourse) [1,13]. The refusal of clients to use condoms and their willingness to pay more for unprotected sex is the greatest barrier to safer commercial sex [15,16]. The "Don Juan" preventive action described here was designed to address these issues. Specifically, we wished to determine whether it was feasible in the red light district of Lausanne, Switzerland to test the clients of sex workers for HIV using rapid testing on finger-stick blood samples. We assessed whether clients would be willing to undergo such testing and determined HIV prevalence in this sample of clients.

## Methods

The study took place over 5 nights in September 2008 (from 10 pm to 1 am) in the red light district of Lausanne, Switzerland. This well defined area consists of 4 parallel streets each less than 400 meters long restricted by law (area and time period). Clients of sex workers were stopped in their cars. A free condom was offered by trained field staff members through the car-window as a means of introduction to an interview. A face to face questionnaire was administered by the trained field worker that focused on age, country of origin, profession, HIV testing history, whether the client had a stable partner or partners, and use of condoms with sex workers. The clients were then offered a free anonymous rapid HIV test (Determine™ HIV-1/2) on finger-stick blood sample that could be performed in a bus parked nearby. Rapid HIV testing and counselling were performed by experienced nurse practitioners in accordance with the Swiss Federal Office of Public Health recommendations for anonymous voluntary counselling and testing (VCT) [17]. When the test results were available, i.e. about 30 minutes after the test was administered, the nurse practitioner communicated the test result to the client (in the bus). The client was offered confirmatory testing, medical evaluation, and care in our HIV clinic if the test result was positive.

The study protocol was approved by the Medical Ethics Committee of the University of Lausanne, and the study was performed in compliance with the Helsinki Declaration.

## Results

**Figure 1:**
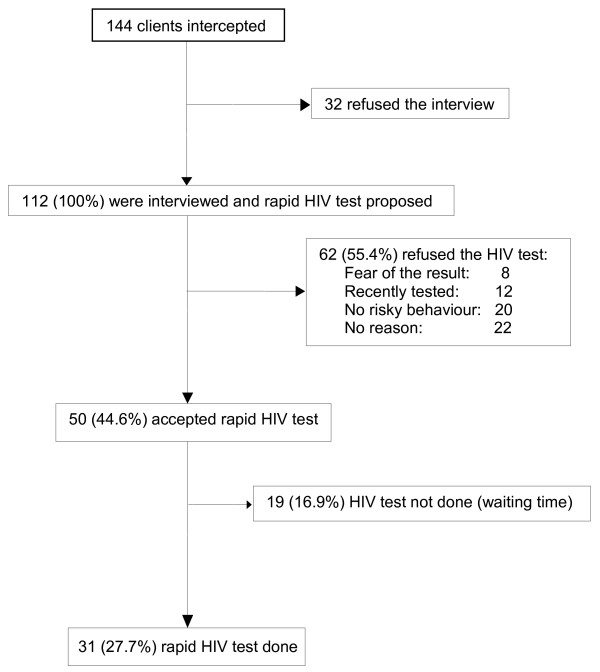
**Flowchart of clients intercepted**.

The clients we stopped were all men who ranged from 19 to 63 years old. Most were from European countries and 40% were in a stable relationship. Clients' characteristics are summarized in Table 1. Between 20 and 60 clients visit the area every night. The general flow of clients intercepted, interviewed and tested for HIV is summarised in figure 1. Of 144 individuals intercepted on the street, 112 (77.8%) agreed to be interviewed. The remaining 32 clients, just took the condom that was handed through the car-window and left without accepting the interview. In the 112 interviewed clients sample, 50 (46.6%) had never had an HIV test, 16/112 (14.3%) did not always use a condom with the sex worker, 45/112 (40.2%) had a stable partner. Of the 31 rapid HIV tests performed (27.7% of the interviewed clients), none were reactive. Of note, 7 of the tested clients (22.6%) did not always use a condom with sex workers. Nineteen additional clients (16.9%) initially agreed to HIV testing but later declined due to delay in testing. Characteristics of the 31 clients who were tested for HIV compared to the 112 clients who were not tested are summarized in Table 1.

**Table 1:** Clients' characteristics: sociodemographic, condom use with sex workers and past HIV tests

		Total = 112	Clients untested n = 81	Clients tested n' = 31
**Age**				
	Mean	36	36	36
	Range	19-63	19-60	19-63
	Missing	0		
**Origin**				
	Swiss	37 (33.1%)	25 (30.9%)	12 (38.7%)
	European community	47 (41.9%)	33 (40.7%)	14 (45.2)
	Others	15 (13.4%)	12 (14.8%)	3 (9.7%)
	Missing	13 (11.6%)	11 (13.6%)	2 (6.4%)
**Stable relationship**				
	Yes	45 (40.2%)	33 (40.8%)	12 (38.7%)
	No	38 (33.9%)	27 (33.3%)	11 (35.5%)
	Missing	29 (25.9%)	21 (25.9%)	8 (25.8%)
**Persistent use of condom with sw^1^**				
	Yes	84 (75%)	60 (74.1%)	24 (77.4%)
	No	16 (14.3%)	9 (11.1%)	7 (22.6%)
	Missing	12 (10.7%)	12 (14.8%)	0
	If no, type(s) of unprotected sexual intercourse(s)^2^:			
	Oral	10	7	3
	Genital	4	2	2
	Anal	2	2	0
	Missing	4	0	4
**HIV test performed**				
	Never	50 (44.6%)	34 (42%)	16 (51.6%)
	within the last 6 months	15 (13.4%)	13 (16.1%)	2 (6.5%)
	within the last 2 years	24 (21.4%)	20 (24.7%)	4 (12.9%)
	more than 2 years ago	15 (13.4%)	10 (12.3%)	5 (16.1%)
	Missing	8 (7.2%)	4 (4.9%)	4 (12.9%)

^1^**sw: **sex worker, ^2^A client could report more than one type of unprotected sexual intercourse

## Discussion

This pilot study showed the feasibility of rapid HIV testing in the red light district of Lausanne. It also revealed an unexpectedly high rate of HIV testing acceptance (i.e. 44.6%) by the clients of sex workers.

To our knowledge, this is the first study to assess the feasibility and acceptance of rapid HIV testing of sex workers' clients in a western country. By placing the bus on a dead end street, the survey and HIV testing took advantage of the well restricted area where cars engaging in the street know that it leads "only" to sex workers. We were able to cover 100% of the cars engaging on this street and approximately 70% of the clients visiting the district (as the vast majority engage on all 4 streets when looking for a street sex worker). The setting of the bus parked in the red light district with professional nurses face to face with the client inside the bus to perform HIV rapid testing was satisfactory from the technical, medical and patient point of view. This setting seems particularly appropriate for targeted HIV screening, since more than 40% of the clients had never been tested for HIV even though they engaged in sex-related HIV risk behaviours. Although sample sizes are small in this pilot study (table 1), we did not find any sociodemographic factors that significantly differ between the clients that accepted or refused the HIV test.

Surprisingly, meeting the client while he was on his way to seek sex with a sex worker allowed a simple, direct, and authentic discussion. The clients' socio-demographic characteristics indicated that they had access to health care and enough money to get tested in anonymous VCT centers. However, more than 50% who were tested on the street had never been tested previously, raising questions about the barriers to HIV testing in VCT or primary care physician settings. Nevertheless, HIV testing on the street allowed us to reach men at high risk for HIV who do not use traditional HIV clinics and who thus merit further study [18]. Some clients acknowledged that our "fly-on-the-wall" approach was effective because this setting allowed them to take an HIV test without justification. The anonymity of the test was also an important factor in clients' decisions to be tested [19]. Rapid HIV testing produces a result in about 30 minutes, and the rapidity is known to facilitate clients usage [19,20]. We do not know whether the fact that the testing was provided for free (compared to 30 euros in the VCT centers) contributed to the high acceptance rate. Studies proposing HIV tests may be confounded by selection bias. The main pitfall is that people who accept the HIV test are the ones who do not need it (because they are at a low risk). Although the sample size of our pilot study is small, we did not find a sociodemographic bias suggesting that the clients who accepted the HIV test were at a lower risk than the clients who refused the HIV test.

This pilot study identified some factors that decreased the test acceptance rate. More than one third of the clients who initially agreed to HIV testing were ultimately not tested owing to an unacceptable waiting time. Indeed, there were more clients willing to get tested for HIV than we anticipated; by having more nurse practitioners and thus decreasing the waiting time, 19 additional HIV rapid tests could have been administered, increasing the number of tests performed to 50 (44.6%). The cold and rainy weather on the 5 autumn nights certainly contributed to clients' reluctance to wait on the street for an HIV test, which is something that should be considered in future studies or when devising a similar HIV testing strategy.

## Conclusion

HIV testing in anonymous VCT centers or caregiver facilities fails to reach a high percentage of the clients of sex workers. HIV testing in red light districts should be considered, since this study shows that such testing is feasible. The cost effectiveness must be assessed, taking into account that late diagnosis of HIV is costly and that early identification of HIV may decrease the spread of the disease to both sex workers and to the clients' regular partners.

## Abbreviations

STD: sexually transmitted disease; VCT: voluntary counselling and testing.

## Competing interests

The authors declare that they have no competing interests.

## Authors' contributions

EAD, AAP, PB and MC contributed to the conception and design of the study. EAD, AAP and CNG collected datas. EAD conducted the statistical analysis and interpreted the data with PB and MC. EAD drafted the manuscript. PB, MC, MV, EM, CNG read, commented and approved the protocol and the manuscript. All authors read and approved the final manuscript.

## Pre-publication history

The pre-publication history for this paper can be accessed here:

http://www.biomedcentral.com/1471-2334/10/74/prepub
